# Knowledge, attitudes, and practices of nurses towards mobile and digital health information tools in Ujjain, India

**DOI:** 10.1177/20552076261469348

**Published:** 2026-07-15

**Authors:** Shweta Khare, Ashish Pathak, Devendra Baghel, Jitendra Jat, Manju Purohit, Salla Atkins, Vinod Kumar Diwan

**Affiliations:** 1 Department of Global Public Health, Health Systems and Policy (HSP): Medicines, Focusing Antibiotics, 27106 Karolinska Institute, Stockholm, Sweden; 2 Department of Public Health and Environment, 78280 Ruxmaniben Deepchand Gardi Medical College, Ujjain, Madhya Pradesh, India; 3 Department of Pediatrics, 78280 Ruxmaniben Deepchand Gardi Medical College, Ujjain, Madhya Pradesh, India; 4 Department of Pathology, 78280 Ruxmaniben Deepchand Gardi Medical College, Ujjain, Madhya Pradesh, India; 5 Department of Global Public Health, Social Medicine, Infectious Diseases and Migration (SIM), 27106 Karolinska Institute, Stockholm, Sweden; 6 WHO Collaborating Centre on Health in All Policies and Social Determinants of Health, Department of Health Sciences, Faculty of Social Sciences, Tampere University, Tampere, Finland; 7 Department of Global Public Health, 27106 Karolinska Institute, Stockholm, Sweden

**Keywords:** digital health, nurses, mobile health applications, maternal health, India

## Abstract

**Background:**

Reliable access to evidence-based clinical information is essential for improving maternal and newborn care, yet nurses in many low-resource settings rely on unstructured digital sources. This study assessed nurses’ knowledge, attitudes, and practices regarding smartphones, internet use, and mobile health applications to inform the development of a maternal–newborn health mHealth intervention.

**Methods:**

A prospective cross-sectional survey was conducted among 453 nurses from three hospitals in Ujjain, Madhya Pradesh, India. A structured, pilot-tested questionnaire measured digital access, information-seeking practices, and attitudes toward mobile healthcare applications. Descriptive statistics were used to summarize findings.

**Results:**

Digital access was high, with 99% of nurses using smartphones and the internet. Most nurses searched online for disease symptoms (97%) and treatment information (91%), primarily through personal mobile data. Only 6% had installed mobile healthcare applications, but 98% expressed willingness to use a credible, evidence-based app. Google and YouTube were the most common search engines used to locate health-related information, and 88% participated in informal social media groups to seek clinical advice. Lack of awareness was the main barrier to using mHealth applications.

**Conclusion:**

Nurses demonstrate strong digital engagement and high readiness to adopt structured mobile healthcare applications, despite limited current use. These findings underline the need for reliable, evidence-based digital tools to support clinical decision-making and reduce reliance on unverified online sources. The study provides essential baseline insights for designing a maternal–newborn mobile health application tailored to the digital behaviors and information needs of frontline nurses.

## Introduction

The COVID-19 pandemic exposed critical vulnerabilities in health systems and highlighted the need for effective mechanisms to support frontline healthcare providers while maintaining the quality and safety of care.^1,2^ During this period, digital health technologies gained importance as tools to facilitate communication, improve access to clinical guidance, and support evidence-based decision-making.^2,3^ As these technologies become increasingly integrated into healthcare delivery, understanding how nurses use and perceive digital health tools has become essential for strengthening clinical practice and supporting informed patient care.^2,4^ Within this evolving digital health landscape, mobile health (mHealth) technologies have emerged as particularly accessible and scalable tools to support healthcare providers in their daily clinical work.^3,4^ In this context, mHealth technologies—including smartphones, messaging platforms, and mobile applications—have emerged as important tools for improving communication, expanding access to clinical guidelines, and offering real-time support to healthcare workers.^5–8^ mHealth is broadly defined as the use of mobile and wireless technologies to support health objectives, including clinical care, health education, and health system strengthening.^7,8^ Informal use of mobile phones is already widespread among healthcare staff in many low- and middle-income countries,^9^ suggesting an opportunity to leverage familiar technologies to strengthen healthcare delivery.

Nurses represent the largest group of healthcare providers and play a central role in patient care delivery, clinical monitoring, and implementation of treatment plans.^10,11^ Their responsibilities often require timely access to accurate clinical information to support safe and effective patient care.^12^ Previous research has shown that nurses frequently use personal smartphones and internet resources to obtain clinical information,^9,13^ communicate with colleagues,^9^ and support patient management.^12^ Despite increasing digital access, the extent to which nurses are prepared to use structured digital health tools in their routine clinical practice remains insufficiently understood, particularly in low- and middle-income countries.

Understanding nurses’ knowledge, attitudes, and practices related to digital health technologies is important for identifying gaps in digital readiness and informing the development of effective digital interventions.^14,15^ Assessing how nurses currently search for health information, their level of trust in digital sources, and their willingness to adopt mobile health applications can help guide the design of tools that are both acceptable and feasible in real-world healthcare settings.^16,17^

Although there is compelling evidence supporting the effectiveness of simple and cost-efficient maternal and neonatal health interventions, their consistent implementation in routine clinical practice remains inadequate in many settings.^18,19^ This gap is influenced by multiple factors including limited clinical knowledge,^20^ inadequate community engagement,^21^ shortages of trained staff,^22^ heavy workloads,^23^ and broader health system challenges that affect the delivery of quality care.^24^ In India, maternal and child health indicators remain an important public health concern,^25,26^ particularly in states such as Madhya Pradesh.^13^

This study was conducted as part of a larger project aimed at developing a mobile health application to support evidence-based maternal and newborn care. However, the present study focused on nurses from all clinical departments to understand general digital information practices across the nursing workforce rather than only those working in maternal or neonatal units. This approach recognizes that nurses across departments frequently rely on digital information sources and may also contribute to maternal or neonatal care through shared responsibilities, staff rotation, or emergency support.

Therefore, the aim of this study was to assess nurses’ knowledge, attitudes, and practices regarding the use of smartphones, internet resources, social media, and mobile healthcare applications. The findings are intended to provide baseline evidence on digital readiness among nurses and to inform the development of a context-appropriate mHealth intervention.

## Methods

### Study design

This study employed a prospective cross-sectional design. The study was carried out from June to September 2023 in Ujjain district, Madhya Pradesh (MP), India. The design was selected to generate baseline information needed to guide the development of a maternal and newborn health mobile application for frontline healthcare workers.

### Study setting

Ujjain is located in the central Indian state of MP, one of India’s larger states both in geographical area and population size. Maternal health indicators in MP rank among the poorest in the country.^25^ As an Empowered Action Group state, MP exhibits alarming health indicator figures, including high neonatal mortality rate (29/1000 live births versus national average of 25/1000 live births,^27^ infant mortality rate (40/1000 live births versus national average of 26/1000 live births),^26^ and under-5 mortality rate (57/1000 live births versus national average of 41/1000),^27^ as well as the third-highest maternal mortality ratio (173/100,000 live births versus national average of 88/100,000 live births)^25^ among the Indian states. According to the National Family Health Survey (NFHS)-5 (2019-21), 25% of women in MP encountered at least one complication during delivery.^27^ The study was conducted across three purposively selected hospitals: RD Gardi Medical College, a 560-bed tertiary teaching hospital; Ujjain District Hospital, a secondary-level government facility; and Ujjain Civil Hospital, another government-run healthcare centre.

These hospitals collectively serve a mixed urban–rural population and employ nurses across diverse specialties, including maternal and child health, internal medicine, surgery, paediatrics, emergency services, and critical care. Including nurses from various departments allowed the study to capture a comprehensive picture of digital readiness across the nursing workforce, acknowledging that nurses may rotate between departments or support maternal-newborn emergencies even when not working in designated obstetric units.

### Study population and eligibility criteria

All registered nurses, regardless of their clinical specialty, age, or years of experience, were eligible to participate. A complete list of nurses working in the three participating hospitals was obtained from the nursing administration prior to data collection. This list served as the sampling frame and included nurses from all departments and all duty shifts. Research assistants visited each hospital in person and approached nurses during their scheduled working hours. Recruitment was carried out systematically across morning, evening, and night shifts to ensure inclusion of nurses with varied schedules and responsibilities.

Nurses were first briefed on the purpose of the study, the voluntary nature of participation, and the confidentiality of their responses. Those who agreed were invited to a quiet area of the ward or nursing station to provide written informed consent. Nurses who were on annual leave, sick leave, or maternity leave during data collection were excluded because they were not available for the consent process or questionnaire administration.

**Figure 1.**
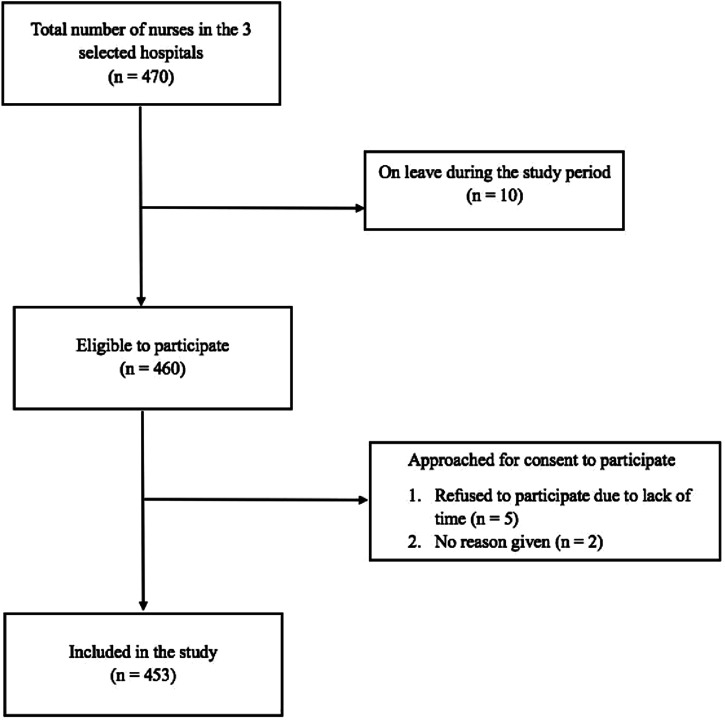
Flow chart of recruitment of participants.

Figure 1 presents the recruitment flow of the 470 nurses listed across the three hospitals, 10 were not available due to leave and could not be contacted. Among the remaining 460 eligible nurses, 7 declined participations—five due to lack of time and two without giving a reason. No additional exclusions were applied. This resulted in a final sample of 453 participants, reflecting a response rate of 98%.

### Sample size calculation

Sample size was calculated using an estimated population of approximately 4,000 nurses employed in both public and private healthcare facilities in Ujjain district. A pilot survey conducted among 50 nurses showed that 25% used the internet to access health-related information while working with patients. The sample size was calculated using the standard formula for estimating a proportion:

$$n=\left(Z^{2}\times p\times \left(1\;‐\;p\right)\right)\;/\;d^{2}$$

where n is the required sample size, Z is the standard normal variate corresponding to the desired confidence level (2.576 for 99% confidence), p is the expected proportion (0.25 based on pilot data), and d is the acceptable margin of error (0.05). Using this estimate, a sample size of 444 participants was required to achieve a 99% confidence level and 80% power, with a margin of error of ±5%. The final sample of 453 nurses exceeded this requirement.

### Data collection tool

Data were collected using a structured questionnaire (Supplementary Table 1) developed following a literature review on digital technology use among healthcare providers. The questionnaire design was informed by previous studies examining healthcare workers’ use of mobile devices, digital information sources, and adoption of digital health tools in clinical practice.^9–13^

The questionnaire underwent pilot testing with 50 nurses not included in the main study to ensure clarity, relevance, and contextual suitability. Content validity was assessed through expert review by senior researchers in public health and digital health, who evaluated the relevance, clarity, and appropriateness of the items. Minor modifications were made to improve wording and comprehension based on their feedback.

Reliability was assessed during the pilot phase by evaluating the internal consistency of the attitude-related items. The questionnaire also underwent face validity assessment to ensure that questions were understandable and reflected real-world information-seeking practices of nurses. Revisions were made based on pilot feedback. The final questionnaire consisted of two major sections: *Sociodemographic characteristics*: age, gender, education, and type of healthcare facility; *Knowledge, attitudes, and practices questions related to*: smartphone ownership and use; internet access and purposes of use; participation in social media groups involving healthcare peers; familiarity with mobile healthcare applications; perceived credibility of online information sources; attitudes toward adopting healthcare mobile applications. A total of 14 items assessed digital knowledge and practices, and 5 items assessed attitudes, including perceived utility and trust in digital platforms.

### Data collection procedure

Trained research assistants conducted one-on-one, paper-based surveys with nurses across all three shifts (8 am–2 pm, 2 pm–8 pm, and 8 pm–8 am) to ensure complete coverage of the nursing workforce. A team of three trained research assistants conducted the data collection over the four-month study period (June–September 2023). Data collection was organized in coordination with nursing supervisors to minimize disruption to clinical duties. Each questionnaire required approximately 10–15 minutes to complete. The survey was conducted in Hindi, the local working language, to support comprehension. Responses were later translated into English for analysis. Completed questionnaires were checked daily for accuracy and completeness. Any missing responses were clarified through follow-up visits to the participants.

### Data management and statistical analysis

Data were double-entered into EpiData Entry v3.1 (EpiData Association, Odense, Denmark) to minimize entry errors. Discrepancies between datasets were resolved through verification against the original paper forms.

Statistical analysis was performed using Stata version 13.0 (StataCorp, College Station, Texas, USA). Descriptive statistics were used to summarize sociodemographic characteristics and digital health practices. Continuous variables are presented as mean ± standard deviation or median with interquartile range (IQR), depending on distribution. Categorical variables are presented as frequencies and percentages.

Daily internet use was categorized into quartiles based on the distribution of reported usage time to facilitate comparison across levels of digital engagement [Q1 (<120 minutes), Q2 (120 - 180 minutes), Q3 (181 - 240 minutes) and Q4 (>240 minutes)). Associations between categorical variables and mobile healthcare application use were assessed using the chi-square test or Fisher’s exact test where appropriate. The dependent variable was use of a mobile healthcare application (yes/no). Independent variables included age category (<30 years and >30 years), gender, healthcare facility type, daily internet use, and participation in social media platforms (included YouTube, WhatsApp, Instagram, Facebook, and Twitter/X). Variables demonstrating a statistically significant association (p < 0.05) in the unadjusted analysis were entered into a multivariable logistic regression model to identify independent predictors of mobile healthcare application use. Adjusted odds ratios (aORs) with 95% confidence intervals (CIs) were reported.

To reduce the risk of type I error arising from multiple comparisons, Bonferroni correction was applied where appropriate. A two-sided p-value of <0.01 was considered statistically significant after adjustment.

### Ethical consideration

The study was approved by the ethics committee of R. D. Gardi Medical College, Ujjain (approval number 27/2022). Written informed consent was taken from the nurses after informing them about the purpose of the study prior to data collection. All responses were anonymized, and data were stored securely with restricted access. It was made clear to them that their participation was voluntary and that all information collected from them would be kept confidential. The procedures followed were in accordance with the ethical standards set by the Institutional ethics committee and the declaration of Helsinki.

## Results

A total of 453 nurses completed the survey. Most participants were women (n = 366, 81%), and the remaining were men (n = 87, 19%). The participants’ ages ranged from 18 to 65 years, with the mean ± SD of 27 ± 8 years. Age distribution showed that most participants were young nursing staff. Most nurses (68%) were employed in private healthcare facilities, while 32% worked in government settings.

### Digital access and internet use (table 1)

Almost all participants (99.7%) owned a mobile phone, and 99% reported using smartphones. Internet use was similarly widespread, with 99% (n = 449) regularly accessing the internet. Internet expenditure ranged from Indian Rupees (INR) 120 to INR 1500 per month (mean: INR 287 ± 128; approximately $1.40 to $17.51; median: INR 250; interquartile range [IQR]: INR 200–350). Daily internet use varied from 20 minutes to 12 hours, with an average of 3 ± 1 hours per day (median: 3 hours; IQR: 2–4 hours). Educational purposes (94%), health-related information (98%), and entertainment (96%) were the most frequent reasons for using the internet (Table 1).

**Table 1.** Knowledge and practices of nurses regarding the use of the internet, smartphones, social media platforms, and mobile healthcare applications (n = 453).

Knowledge and practice	n (%)
1. Use of internet	449 (99)
2. Availability of internet without cost at workplace	5 (1)
3. Access the internet using personal mobile data	333 (74)
4. Using colleague’s/friend’s mobile phone data	179 (40)
5. Reasons for using internet:	
a) Education	428 (94)
b) Collecting health-related information	442 (98)
c) Entertainment	436 (96)
6. Presence on informal social media (WhatsApp, Signal, Telegram) groups along with peers/other nurses/doctors?	395 (88)
7. Main purpose of forming or being part of the informal social media (WhatsApp, Signal, Telegram) groups	
a) To get/share advice on patients’ condition/illness	221 (49)
b) For sharing information related to the patient (treatment, reports)	181 (40)
e) Sharing updates and links about new developments in the healthcare field	136 (30)
f) To get the emotional support of peers	110 (24)
g) For sharing informal messages	54 (10)
1. Any mobile healthcare application on participants’ phones	27 (6)
2. Training on the use of mobile healthcare applications	4 (15)
3. Search for the signs/symptoms of any disease in a patient	435 (97)
4. Search for disease-specific treatments	408 (91)

### Use of social media and information-seeking behavior (table 1)

A large proportion of nurses (88%) reported participation in informal social media groups (such as WhatsApp, Signal, and Telegram) involving peers, other nurses, or doctors. These groups were commonly used to seek or share advice on patient conditions (49%), share investigation reports or treatment-related information (40%), exchange updates on new developments in healthcare (30%), or seek emotional support (24%). None of the nurses reported participation in formal, institutionally supported professional groups.

Although only 6% of participants had mobile healthcare applications installed on their devices, 97% reported using the internet to search for signs or symptoms of diseases, and 91% used it to search for disease-specific treatment information.

### Perceived credibility of online information (table 2)

Google (95%) and YouTube (34%) were the most commonly trusted search engines used to locate sources providing health-related information, while a smaller proportion relied on platforms such as WhatsApp (4%) or other social media sites. Most participants (83%) agreed or strongly agreed that the information they accessed through these platforms was credible (Table 2).

**Table 2.** Nurses attitude towards the usefulness and credibility of mobile applications providing health-related information (n = 449).

Attitude	n (%)
1. Which among the following is the most trusted platform for healthcare- related information according to you?	
a) Google	426 (95)
b) YouTube	151 (34)
c) WhatsApp	20 (4)
d) Others (Instagram, Facebook, Twitter)	14 (3)
2. Do you agree that the aforementioned platforms provide credible healthcare-related information?	
a) Agree	371 (83)
b) Fully Agree	68 (15)
c) Disagree	10 (2)
3. Reasons for not using mobile healthcare applications:	
a) Not aware about such applications	243 (54)
b) Do not use mobile healthcare applications despite being aware	97 (22)
c) Unaware of the process to use the applications	59 (13)
d) Lack of trust on such applications	22 (5)
e) Afraid of getting cheated while using such applications	14 (3)
f) Afraid of breach of personal information while using such applications	14 (3)
4. How important are mobile healthcare applications in the continuous improvement of the quality of healthcare?	
a) Important	308 (69)
b) Very important	119 (27)
c) Less important	22 (5)
5. If a mobile healthcare application is provided to you, will you use it?	
a) Yes	439 (98)
b) No	10 (2)

### Attitudes toward mobile health applications (table 2)

Most nurses (69%) considered mHealth applications important for improving the quality of healthcare, and 98% expressed willingness to use a mHealth application if provided. The most frequently reported barrier to current mHealth application use was lack of awareness (54%), followed by lack of knowledge on how to use such applications (13%) and concerns about trust or data privacy (less than 5%).

### Factors associated with mobile healthcare application use (table 3)

Inferential analysis was conducted to identify factors associated with the use of mobile healthcare applications among nurses. In the unadjusted analysis, younger age, male gender, higher daily internet use, and participation in social media platforms were associated with mobile healthcare application use (Table 3).

**Table 3.** Factors associated with mobile healthcare application use among nurses.

Variable	Total (n=453)	Mobile HealthCare application use		OR (95% CI)	p-value	Adjusted OR (95% CI)	p-value
		Yes (n = 27, 6%)	No (n = 426, 94%)				
**Age Categories**							
<30 years	102	11 (11)	91 (89)	R	-	-	-
≥30 years	351	16 (5)	335 (95)	0.39 (0.17 – 0.88)	0.023^#^	0.34 (0.14 – 0.78)	0.012
**Gender**							
Female	366	15 (4)	351 (96)	R	-	-	-
Male	87	12 (14)	75 (86)	3.74 (1.68 – 8.32)	0.001^#^	4.09 (1.78 – 9.36)	0.001
**Facility type**							
Private	312	15 (5)	297 (95)	R	-	-	-
Public	141	12 (9)	129 (91)	1.84 (0.83 – 4.04)	0.128	-	-
**Daily internet use (n = 449)** *							
Q1 (<120 minutes)	158	7 (4)	151 (96)	R	-	-	-
Q2 (120 - 180 minutes)	133	7 (5)	126 (95)	1.19 (0.40 – 3.50)	0.741	-	-
Q3 (181 - 240 minutes)	95	5 (5)	90 (95)	1.19 (0.36 – 3.88)	0.763	-	-
Q4 (>240 minutes)	63	8 (13)	55 (87)	3.13 (1.08 – 9.05)	0.035	-	-
**Use of social media platforms**							
Non-users	286	10 (4)	276 (96)	R	-	-	-
Users	167	17 (10)	150 (90)	3.12 (1.39 – 7.00)	0.006**^#^**	3.25 (1.42 – 7.43)	0.005

Notes: Data are presented as n (% row percentage).

*Daily internet use was assessed only among participants who reported using the internet (n = 449). Social media Platforms distribution among users included YouTube, WhatsApp, Instagram, Facebook, and Twitter/X.

^#^Adjusted odds ratios are presented only for variables that showed a statistically significant association (p < 0.05) in the unadjusted analysis and were subsequently included in the multivariable logistic regression model.

In the multivariable logistic regression model, male nurses were more likely to report mobile healthcare application use than female nurses (aOR 4.09; 95% CI 1.78–9.36; p = 0.001). Participation in social media platforms also remained independently associated with mobile healthcare application use (aOR 3.25; 95% CI 1.42–7.43; p = 0.005).

A lower likelihood of mobile healthcare application use was observed among nurses aged over 30 years compared with younger nurses (aOR 0.34; 95% CI 0.14–0.78; p = 0.012); however, this association did not meet the predefined significance threshold following Bonferroni adjustment. Higher daily internet use was associated with mobile healthcare application use in the unadjusted analysis, but this association was not retained in the multivariable model. Facility type was not significantly associated with mobile healthcare application use. These findings suggest that demographic characteristics and digital engagement behaviours are associated with mobile healthcare application use among nurses.

## Discussion

This study provides an in-depth understanding of nurses’ knowledge, attitudes, and practices related to digital technologies in healthcare, with relevance for supporting maternal and newborn care. The findings demonstrate that while digital device penetration is nearly universal among nurses, the use of structured mHealth applications remains low. Instead, nurses rely heavily on general internet searches and informal social media groups to access information related to patient conditions, treatments, or clinical decision-making.^9^ These patterns reflect both the opportunity and the challenges involved in designing digital tools that can integrate effectively into everyday nursing practice.

The high prevalence of smartphone ownership and frequent internet use among nurses aligns with previous research highlighting the increasing role of mobile technology in healthcare delivery and communication.^5–9,13^ However, much of this engagement occurs through unstructured information-seeking behavior. In this study, nurses frequently searched online for disease symptoms (97%) and treatments (91%) during clinical encounters, yet only 6% had installed mHealth applications. This suggests that while mHealth tools are not yet part of routine practice, there is clear potential for adoption, supported by the finding that 98% of nurses expressed willingness to use a structured, evidence-based application if available.

The inferential analysis provided additional insight into factors associated with mobile healthcare application use among nurses. Male nurses were more likely to report mobile healthcare application use than female nurses. Although evidence regarding gender differences in digital health adoption among nurses remains limited, previous studies have reported variations in technology use, digital confidence, and engagement with digital resources among healthcare professionals which may contribute to differences in the uptake of digital tools.^4,17^ However, the reasons underlying this difference remain unclear and may reflect contextual, organizational, or behavioural factors that were not measured in the present study.

A more notable finding was the independent association between participation in social media platforms and mobile healthcare application use. Nurses who actively engaged with social media were more likely to report using mobile healthcare applications, suggesting that familiarity with digital communication platforms may reflect broader digital readiness and willingness to engage with technology-enabled healthcare solutions. This finding is consistent with previous evidence showing that healthcare workers frequently use mobile phones and online platforms for communication, information seeking, and professional support, and that familiarity with digital technologies can facilitate the adoption of new digital health tools.^28^ Importantly, this finding suggests that engagement with digital platforms may serve as a practical indicator of digital readiness, reflecting both familiarity with technology and willingness to incorporate digital tools into professional activities.^14,17^

Although nurses aged over 30 years showed a lower likelihood of mobile healthcare application use in the adjusted analysis, this association did not meet the predefined significance threshold following Bonferroni adjustment. Nevertheless, the finding may reflect differences in exposure to and familiarity with digital technologies across age groups and warrants further investigation in larger studies.

Overall, the inferential findings complement the descriptive results by demonstrating that digital engagement behaviours, particularly participation in social media platforms, are associated with greater readiness to use mobile healthcare applications than demographic characteristics alone. These findings highlight the importance of considering digital readiness during the design and implementation of future mHealth interventions.^4,28^

An important concern is the reliance on general search engines such as Google and YouTube to locate health-related information. These platforms are easily accessible and familiar, making them attractive in time-constrained clinical settings, but they do not provide controlled or context-specific clinical guidance. Previous literature has similarly highlighted concerns regarding the variable accuracy of online content and the risks of relying on non-curated sources for clinical decision-making.^12^ This is particularly important in maternal and newborn care, where timely recognition of danger signs is critical.^18,19^ Together, these findings emphasize the need for reliable, evidence-based digital tools that can support clinical decision-making and reduce dependence on general internet searches and informal peer networks.

The widespread use of informal social media groups among nurses further illustrates unmet needs for rapid support and access to expertise. Nurses frequently used platforms such as WhatsApp, Signal, and Telegram to discuss clinical concerns, share reports, and seek guidance from colleagues. While previous evidence shows that healthcare workers often rely on informal mobile-phone–based communication to support clinical tasks,^9^ such communication lacks standardization and quality control. Strengthening digital support through trustworthy, validated tools could provide greater safety and consistency.

Artificial intelligence (AI)-enabled mHealth applications can offer features such as automated triage suggestions, context-specific alerts, rapid access to guidelines, and personalized educational content.^5,6,29^ Although the nurses in this study were not using AI-supported tools, their strong willingness to adopt credible mHealth applications suggests that future AI-integrated maternal health tools could be feasible. Understanding current digital behaviors therefore provides the necessary foundation for designing such advanced systems in a way that aligns with local digital practices, infrastructure limitations, and user expectations.

Taken together, the descriptive and inferential findings suggest that the foundation for digital health adoption is strong: nurses possess smartphones, routinely engage with the internet, and demonstrate clear motivation to access reliable information given the information-intensive nature of nursing practice.^30^ The challenge lies in the absence of institutionally supported, evidence-based mHealth tools and the lack of training on their use. Addressing these gaps may substantially improve access to maternal and newborn health guidelines, reduce variability in information quality, and strengthen clinical decision-making.

This study is both necessary and innovative for several reasons. First, it captures digital readiness not only among maternal-health nurses but across the entire nursing workforce, reflecting real-world staffing dynamics where skills and responsibilities overlap across departments.^12,29,30^ Second, the survey instrument used in this study was explicitly designed to assess knowledge, attitudes, and practices related to health-information seeking, including how nurses look for disease symptoms, treatment options, clinical updates, and professional peer support. Unlike general internet-use surveys, this questionnaire was anchored in the specific needs of maternal and newborn health. It captured digital behaviors that directly influence clinical safety—such as reliance on online searches, lack of access to formal guidelines, and trust in uncurated content. Because of this, the findings provide crucial insights for designing a maternal–newborn mHealth intervention that is aligned with genuine clinical information needs, workflow realities, and user behavior patterns.

### Practical implications and recommendations

The findings suggest several practical steps to support the use of digital tools in nursing practice. Hospitals could introduce approved mobile health applications that provide reliable clinical guidance and reduce dependence on general internet searches. Providing institutional internet access and short training sessions on the use of digital health tools may improve adoption and effective use. In addition, the association between participation in social media platforms and mobile healthcare application use suggests that existing digital engagement behaviours could be leveraged to support the introduction and uptake of evidence-based mHealth tools. Training should also focus on helping nurses assess the reliability of online health information. Future research should evaluate the usability, acceptance, and impact of maternal–newborn mHealth applications and identify strategies that support their sustained use in routine clinical care.

### Significance of the study

This study contributes to the understanding of digital health adoption by showing that nurses already use digital devices extensively but lack access to structured clinical applications. The findings highlight the importance of integrating reliable mHealth tools into routine nursing workflows rather than introducing them as separate systems. The study also identifies digital engagement behaviours, particularly participation in social media platforms, as potential indicators of digital readiness that may influence future adoption of mHealth applications. By identifying current information-seeking practices and readiness for digital tools, the study provides important evidence for developing mHealth solutions that can support timely decision-making and potentially improve the quality of maternal and newborn care.

## Strengths and limitations

This study has several strengths. It provides a detailed, context-specific assessment of nurses’ knowledge, attitudes, and practices regarding digital information use across multiple clinical departments, rather than limiting the sample to maternal or neonatal units alone. This broad inclusion reflects real patterns of staff rotation and emergency support, enhancing the applicability of the findings for designing district-wide mHealth interventions. The sample size was large, with a response rate of 98%, reducing the likelihood of non-response bias. The questionnaire was pilot-tested and refined before the main study, improving clarity and ensuring that the instrument captured meaningful indicators of digital information-seeking behavior relevant to clinical practice. Although the tool did not specifically focus on maternal or neonatal health information-seeking, it assessed general patterns of accessing clinical information, which are applicable to maternal and newborn care where timely access to reliable information is essential. Data collection was conducted across all nursing shifts, ensuring representation of day, evening, and night staff. The inclusion of inferential analyses further strengthened the study by identifying factors associated with mobile healthcare application use and providing additional insight into nurses’ digital readiness.

Despite these strengths, the study has limitations. As a cross-sectional survey, it reflects nurses’ practices and perceptions at one point in time and cannot establish causal relationships. The findings are based on self-reported digital practices, which may be affected by recall bias or social desirability bias. This risk was reduced by ensuring anonymity, using neutral question wording, and conducting the survey in a private setting. Response bias is also possible, although the high response rate (98%) reduces this concern. In addition, only a small proportion of participants reported using mobile healthcare applications, which limited the number of outcome events available for multivariable analysis and may have reduced the precision of some estimates. The study was conducted in three hospitals within one district, which may limit generalizability to other regions or healthcare systems with different infrastructure or digital policies. However, the inclusion of nurses from multiple departments improves the relevance of the findings for similar hospital settings. These limitations should be considered when interpreting the results but do not affect the study’s value in informing the development of structured mHealth tools.

## Conclusion

This study shows that while nurses in Ujjain have near-universal access to smartphones and the internet, the use of structured mHealth applications is still limited. Despite this low adoption, nurses frequently search for health-related information online and express strong willingness to use a reliable mHealth tool if one were available. Participation in digital communication platforms was also associated with mobile healthcare application use, suggesting that existing digital engagement behaviours may support future adoption of mHealth interventions. These findings indicate a clear need for accessible, evidence-based digital resources that can provide trustworthy clinical guidance, reduce reliance on unverified online content, and support timely decision-making—particularly in maternal and newborn care. The study offers important baseline insights that will directly inform the development of a context-appropriate maternal mHealth application tailored to the digital behavior and practice needs of frontline nurses.

## Supplemental material

Supplemental material - Knowledge, attitudes, and practices of nurses towards mobile and digital health information tools in Ujjain, India Supplemental material for Knowledge, attitudes, and practices of nurses towards mobile and digital health information tools in Ujjain, India by Shweta Khare, Ashish Pathak, Devendra Baghel, Jitendra Jat, Manju Purohit, Salla Atkins and Vinod Kumar Diwan in Digital Health.

## Data Availability

The datasets generated and analyzed during the current study are not publicly available due to institutional data protection policies and the confidentiality of participant information. De-identified data may be made available from the corresponding author upon reasonable request and with approval from the Institutional Ethics Committee of RD Gardi Medical College, Ujjain.*
