# Supplementary material for: Knowledge, attitudes, and practices of nurses towards mobile and digital health information tools in Ujjain, India
Source: Digit Health. 2026 Jul 15;12:20552076261469348. doi: 10.1177/20552076261469348 (PMC13373431; doi:10.1177/20552076261469348)
Supplement: Supplemental material - Knowledge, attitudes, and practices of nurses towards mobile and digital health information tools in Ujjain, India [file sj-pdf-1-dhj-10.1177_20552076261469348.pdf]

Implementation of improved maternal and neonatal care using interactive Intervention with skill teaching videos and real-time supervision and monitoring (MiMhi): A cluster randomized control trial
R.D. gardi Medical College Ujjain, Surasa (M.P.)

Assessment Questionnaire

Unique ID: ____________

Date: ____________

Participant's Name: ____

Age (in years): ____________

Name of Healthcare Facility: ____

Department: _____________________________

Questionnaire to Assess Staff Competency in Using the "Healthcare" Mobile Application

Section I: Respondent Information

1. **Under which category do you work?**
   - A. Nurse (ANM/Nursing Officer)
   - B. ASHA Worker
   - C. Anganwadi Worker
2. **What is your age?**

   ____________ (in years)

3. **Gender**:
   - A. Male
   - B. Female

4. **Which type of healthcare facility do you work in?**
   - A. Private Healthcare Facility
   - B. Public/Government Healthcare Facility
   - C. Both Private and Public/Government Healthcare Facilities

5. **Do you use a mobile phone? (Practice)**
   - A. Yes
   - B. No

6. **What type of mobile phone do you use? (Practice)**
   A. Smartphone
   B. Basic Mobile Phone (only calling facility available)
7. **Do you use the internet? (Practice)**
   A. Yes
   B. No

7a. If yes, what is your monthly expenditure on internet usage? (Knowledge) (Note: Include the total cost of internet connection for both home and mobile.) _____________ (in rupees)

Note: If the respondent does not use the internet, end the interview after Question 7.

8. **Is free internet access available at your workplace? (Practice)**
   A. Yes
   B. No

9. **Which other mediums do you use to access the internet? (Practice)** (Please select one or more options.)
   A. Personal mobile phone
   B. Colleague's/friend's mobile phone
   C. Nearby cyber café
   D. Other: __________________________________
10. **How many hours do you use the internet per day? (Knowledge)**
    A. _____ hours
    B. I do not use the internet.
11. **What is your primary purpose for using the internet? (Practice)**

S.no.	Application Name	Time Spent (Daily)	Purpose of Use			
			Educational Purposes	Health-Related Information	Entertainment	Other
1.	YouTube					
2.	Google					
3.	WhatsApp					
4.	Instagram					

5.	Facebook					
6.	Twitter					
7.	LinkedIn					
8.	Other:_____					

12. **Which social media platforms do you trust most for health-related information?** (Please select one or more options.) **(Attitude)**
    A. YouTube
    B. Google
    C. WhatsApp
    D. Instagram
    E. Facebook
    F. Twitter
    G. LinkedIn
    H. TikTok
    I. Other: ________________________________
13. **How much do you agree with the reliability of mobile healthcare websites providing patient disease-related information? (Attitude)**
    A. Strongly Disagree
    B. Disagree
    C. Agree
    D. Strongly Agree
14. **Are you part of any group on social media platforms (e.g., WhatsApp, Signal, Telegram) with colleagues/other nurses/doctors? (Practice)**
    A. Yes
    B. No
15. **What is your primary purpose for creating or joining groups on social media platforms (e.g., WhatsApp, Signal, and Telegram) with colleagues/other nurses/doctors?** (Please select one or more options.) **(Practice)**
    A. To seek/share advice on patients' conditions/diseases

B. To share patient-related information (treatment, reports)
C. To share updates and links about new developments in healthcare
D. To use the platform for emotional support from colleagues
E. To share informal messages
F. Other: ________________________________

Section II: Information on Mobile Applications Used for Health-Related Information

1. **Do you use the internet to find information about patients' disease symptoms and signs? (Practice)**
   A. Yes
   B. No
2. **Do you use the internet specifically to search for treatments for diseases? (Practice)**
   A. Yes
   B. No
3. **Do you have any healthcare applications on your mobile phone for obtaining patient disease-related information? (Knowledge)**
   A. Yes (please specify the name): ________________________________
   B. No

   3a. If no, why not? (Attitude)
   (Please select one or more options.)
   A. I am not aware of any such applications.
   B. I am aware but do not trust applications providing patient disease-related information.
   C. I am afraid of being scammed while using these applications.
   D. I am concerned about the misuse of my personal information while using these applications.
   E. I am aware of such applications but do not know how to use them.
   F. I am aware of such applications but do not currently use them.

3b.Have you received specific training for using mobile healthcare applications providing patient disease-related information? (Knowledge)

A. Yes
B. No

4. **In your opinion, how important are healthcare mobile applications for continuously improving the quality of healthcare services? (Attitude)**

A. Not Important
B. Slightly Important
C. Important
D. Very Important

5. **If a healthcare mobile application were provided to you, would you use it?(Attitude)**

A. Yes
B. No

For Office Use Only

	Name	Sign	Date
Form filled by			
Form checked by			
Form entered in the data based by			
